# Impact of loneliness on depression among cancer survivors: a comparison between adolescents and young adults and other age groups

**DOI:** 10.1186/s12885-025-14734-4

**Published:** 2025-08-16

**Authors:** Ken Kurisu, Masako Okamura, Keiko Ozawa, Saki Harashima, Kazuhiro Yoshiuchi, Yosuke Uchitomi, Maiko Fujimori

**Affiliations:** 1 Department of Cancer Survivorship and Digital Medicine, Jikei University School of Medicine, Tokyo, Japan; 2 Division of Survivorship Research, National Cancer Center Institute for Cancer Control, 5-1-1 Tsukiji, Chuo-ku, Tokyo, 104-0045 Japan; 3 Department of Stress Sciences and Psychosomatic Medicine, Graduate School of Medicine, The University of Tokyo, Tokyo, Japan

**Keywords:** Adolescents and young adults, Cancer, Loneliness, Depression, Structural equation modeling

## Abstract

**Background:**

Numerous studies have suggested an association between loneliness and depression in cancer survivors, particularly adolescents and young adults (AYAs). This study aimed to develop a causal model linking loneliness to depression using structural equation modeling.

**Methods:**

A cross-sectional web-based survey was conducted to collect demographic information and psychosocial measures, including the UCLA Loneliness Scale, Patient Health Questionnaire-9, EQ-5D-5L, Multidimensional Scale of Perceived Social Support short form, Brief Resilience Scale, Comprehensive Score for Financial Toxicity, and a single item on cancer-related stigma. Structural equation modeling with observed variables was conducted, focusing on the pathway from loneliness to depression. Multiple-group analysis was used to compare AYAs and non-AYAs.

**Results:**

The study included 3,565 cancer survivors, of whom 743 (20.8%) were AYAs. The final model showed a significant association between loneliness and depression (standardized coefficient = 0.214; 95% confidence interval = 0.184–0.243; *P* < 0.001). Perceived social support, resilience, financial toxicity, and stigma were directly or indirectly related to loneliness and depression. Female sex and having a spouse or partner were associated with perceived social support, whereas non-job-related social participation was associated with loneliness. The association between loneliness and depression was significantly stronger among AYAs than non-AYAs.

**Conclusions:**

The results suggested a significant link between loneliness and depression, with a stronger pathway in AYAs. Loneliness may serve as a modifiable mediator for interventions targeting depression prevention among AYA cancer survivors. The psychosocial variables identified could aid in screening high-risk individuals and developing effective interventions.

## Background

People with cancer show high rates of depressive symptoms and suicide [1–3]. Major disease diagnoses increase loneliness [4, 5], and loneliness has an influence on depression [6]. These findings highlight the need to explore the association between cancer, loneliness, and depression.

Cancer in the adolescent and young adult (AYA) population accounts for approximately 4% of all diagnoses in Japan [3]. This life stage has recently been characterized by instability in education, employment, and relationships with friends, partners, and family [7], potentially making AYAs particularly vulnerable to the social impacts of cancer. AYAs with cancer have lower educational attainment, higher unemployment, and lower marriage rates compared to their peers without cancer [8], and they also experience a wide range of adverse health outcomes [9]. They often experience social isolation and loneliness [10], which further correlates with depression [11]. AYA cancer survivors have higher rates of depression and anxiety than their peers without cancer [12, 13]. They also experienced heightened levels of distress during the COVID-19 pandemic [14]. Many AYA survivors also expressed unmet psychological care needs [15], and efforts have been made to develop intervention programs tailored to this generation [16].

These findings suggest an association between loneliness and depression in individuals with cancer, particularly among AYAs who are more socially vulnerable. We hypothesized that modeling this association could enhance the screening of individuals at high risk for depression and help identify components for intervention. Structural equation modeling has been used to investigate the relationship between loneliness and depression, demonstrating cross-sectional and longitudinal associations with psychosocial factors such as social support, stigma, resilience, and quality of life (QOL) [11, 17–20]. However, few studies have investigated generational differences focusing on AYA cancer survivors, likely due to the challenges in conducting research on this relatively rare and geographically dispersed population. Online surveys may offer a feasible approach to addressing this limitation.

This study aimed to develop an exploratory causal model linking loneliness to depression in individuals with cancer and to examine generational differences in the influence of loneliness between AYAs and older adults, using data collected through an online survey.

## Methods

### Ethics approval

This study was approved by the Institutional Review Board of the National Cancer Center, Japan (Approval Number: 2024 − 118). Informed consent was obtained from all the participants.

### Data source

Data were collected through a cross-sectional survey conducted by Macromill, Inc., a Japanese Internet research company with over one million registered members [21]. Eligible participants met the following criteria: (1) aged ≥ 16 years, (2) diagnosed with cancer at age ≥ 15 years, (3) diagnosed within the past 10 years, and (4) had sufficient Japanese proficiency to complete the survey. We requested that approximately 25% of the sample comprised AYA participants. Data collection was conducted between September 19, 2024, and September 29, 2024, with responses obtained only from individuals who provided informed consent.

### Measurements

The web survey collected demographic information, including age, sex, residential prefecture, employment or school status, non-job-related social participation (e.g., volunteering), household income, family status, education, and cancer-related details such as diagnosis time, type, and stage at diagnosis, as well as current ECOG Performance Status [22]. Psychosocial factors were assessed using the following scales:

Perceived loneliness was measured using the Japanese version of the UCLA Loneliness Scale version 3, a 20-item, 4-point self-report scale, with scores ranging from 20 to 80, with higher scores indicating greater loneliness [23].

Depression was evaluated using the Japanese version of the Patient Health Questionnaire-9, a 9-item, 4-point self-report scale, with scores ranging from 0 to 27, with higher scores indicating greater depression [24].

QOL was assessed using the Japanese version of the EQ-5D-5L, a 5-item, 5-point self-report scale, with scores ranging from − 0.111 to 1.000, with higher scores reflecting better QOL [25].

Perceived social support (PSS) was measured using the Japanese short form of the Multidimensional Scale of Perceived Social Support, a 7-item, 7-point scale, with scores ranging from 7 to 49, with higher scores reflecting stronger PSS [26].

Resilience was assessed using the Japanese version of the Brief Resilience Scale, a 6-item, 5-point self-report scale, with scores ranging from 6 to 30, with higher scores indicating greater resilience [27].

Financial toxicity caused by cancer was evaluated using the Japanese version of the Comprehensive Score for Financial Toxicity, an 11-item, 5-point scale measuring financial stress, with scores ranging from 0 to 44 [28]. Lower scores indicate higher financial toxicity. For analysis purposes, the scores were reversed to ensure that higher values represent greater financial toxicity.

Finally, perceived social stigma related to cancer was assessed using a single-item, 4-point Japanese question, with scores ranging from 1 to 4, with higher scores indicating stronger perceived stigma [29].

### Statistical analyses

The associations among the psychosocial scales were explored using structural equation modeling with observed variables (path analysis). We hypothesized that objective isolation would lead to subjective loneliness, which in turn would lead to depression. Accordingly, the model was developed to include a regression path from loneliness to depression.

Additionally, the QOL was included as an outcome influenced by depression. Subjective measurements, including PSS, resilience, financial toxicity, and stigma, were incorporated as variables that directly or indirectly influenced loneliness and depression. Cancer-related factors, such as duration since diagnosis, stage at diagnosis, and ECOG performance status, were included for their potential effects on depression and QOL.

Objective factors, including educational level (university graduate or higher or other), family status (presence of a spouse/partner, children, and caregiving responsibilities), employment/school status, and non-job-related social participation, were also included as variables influencing subjective measurements. These were represented as binary variables (e.g., employed or in school = 1; unemployed or unknown = 0). Sex was included as an adjustment variable for all paths. The standardized mortality ratio for suicide among individuals with cancer, specific to each prefecture, was included as a regional factor [30]. Due to numerous missing answers, household income was excluded from the model. To assess the impact of cancer type, its association with loneliness score was analyzed using a multivariable linear regression model, and inclusion was considered when the regression coefficient was statistically significant.

Based on these considerations, the model was refined by evaluating fit indices, including the comparative fit index (CFI), goodness of fit index (GFI), adjusted GFI (AGFI), and root mean square error of approximation (RMSEA). To maintain model simplicity, scale scores were initially used as observed variables. Latent variables constructed from individual items or subscales were explored when a sufficient fit could not be achieved only with observed variables.

All analyses were performed using R version 4.3.1 (R Foundation for Statistical Computing, Vienna, Austria) and the “lavaan” package version 0.6–16. A p-value < 0.05 was considered statistically significant.

### Group comparison of AYA and non-AYA individuals

To examine generational differences in the impact of loneliness on depression, a multi-group analysis comparing AYAs (≤ 39 years) and non-AYAs (≥ 40 years) was conducted. The standardized regression coefficients for the loneliness-to-depression pathway were evaluated using non-overlapping 95% confidence intervals (CIs) to define significance.

Additionally, a model that assumed equality in the loneliness-to-depression path coefficients for the AYA and non-AYA groups was developed. Subsequently, a chi-square difference test was performed to compare the fitness of this constrained model with that of the unconstrained model.

## Results

### Descriptive statistics

Table 1 shows descriptive statistics for the survey respondents (*N* = 3,565), including 743 (20.8%) AYA participants (< 40 years) and 2,822 (79.2%) non-AYA participants. Table 2 presents the correlation coefficients between the scales, all of which were statistically significant.

**Table 1:** Descriptive statistics

	AYA (*N* = 743)	Non-AYA (*N* = 2822)
Age, mean (SD)	32.2 (6.0)	64.4 (9.6)
Sex, N (%)		
Male	272 (36.6)	1866 (66.1)
Female	471 (63.4)	956 (33.9)
Household income, N (%)		
Under 4 million yen	122 (16.4)	993 (35.2)
4–8 million yen	233 (31.4)	916 (32.5)
Over 8 million yen	205 (27.6)	451 (16.0)
Unknown or not provided	183 (24.6)	462 (16.4)
Education level, N (%)		
University graduate or higher	395 (53.2)	1369 (48.5)
Other	348 (46.8)	1453 (51.5)
Family status, N (%)		
Spouse or partner	477 (64.2)	2236 (79.2)
Children	432 (58.1)	2081 (73.7)
Caregiver responsibilities	175 (23.6)	281 (10.0)
Employment or school, N (%)		
Any job or social role	715 (96.2)	1897 (67.2)
None	28 (3.8)	925 (32.8)
Non-job-related social participation		
Any social participation	441 (59.4)	1159 (41.1)
None	302 (40.6)	1663 (58.9)
Scales, mean (SD)		
UCLA Loneliness Scale	47.5 (10.2)	44.5 (12.2)
Patient Health Questionnaire-9	7.7 (6.0)	3.9 (4.4)
EQ-5D-5L	0.76 (0.19)	0.88 (0.14)
Perceived Social Support	31.7 (10.4)	33.8 (9.4)
Brief Resilience Scale	17.1 (4.7)	19.3 (5.3)
Comprehensive Score for Financial Toxicity	20.7 (7.2)	24.9 (8.6)
Stigma	2.2 (0.9)	1.5 (0.7)
Cancer information		
Time since diagnosis (years), mean (SD)	3.9 (2.9)	4.7 (3.2)
Performance status, mean (SD)	0.5 (0.8)	0.3 (0.5)
Cancer stage		
Stage 0, N (%)	135 (18.2)	420 (14.9)
Stage I, N (%)	321 (43.2)	846 (30.0)
Stage II, N (%)	119 (16.0)	569 (20.2)
Stage III, N (%)	47 (6.3)	355 (12.6)
Stage IV, N (%)	21 (2.8)	211 (7.5)
Unknown stage, N (%)	100 (13.5)	421 (14.9)
Cancer type (multiple choices allowed), N (%)		
Head and neck	39 (5.2)	95 (3.4)
Esophagus	99 (13.3)	99 (3.5)
Stomach	122 (16.4)	342 (12.1)
Small intestine/colon/rectum	114 (15.3)	564 (20.0)
Liver/biliary system/pancreas	73 (9.8)	80 (2.8)
Lung	84 (11.3)	232 (8.2)
Skin	33 (4.4)	53 (1.9)
Breast	116 (15.6)	439 (15.6)
Cervix uteri	134 (18.0)	83 (2.9)
Corpus uteri	47 (6.3)	77 (2.7)
Ovary	45 (6.1)	45 (1.6)
Prostate/testis	35 (4.7)	481 (17.0)
Bladder	24 (3.2)	140 (5.0)
Kidney	26 (3.5)	111 (3.9)
Brain	19 (2.6)	10 (0.4)
Thyroid	63 (8.5)	89 (3.2)
Hematologic cancer	75 (10.1)	149 (5.3)
Others	95 (12.8)	122 (4.3)

*AYA* Adolescents and young adults, *SD* Standard deviation

**Table 2:** Correlation matrix

	Loneliness	Depression	Quality of life	Social support	Resilience	Financial toxicity	Stigma
Loneliness	1						
Depression	0.47**	1					
Quality of life	−0.25**	−0.57**	1				
Social support	−0.68**	−0.36**	0.22**	1			
Resilience	−0.54**	−0.48**	0.28**	0.33**	1		
Financial toxicity	0.40**	0.47**	−0.35**	−0.25**	0.48**	1	
Stigma	0.25**	0.38**	−0.33**	−0.16**	−0.28**	0.31**	1

**p < 0.001

### Structural equation model

**Fig. 1:**
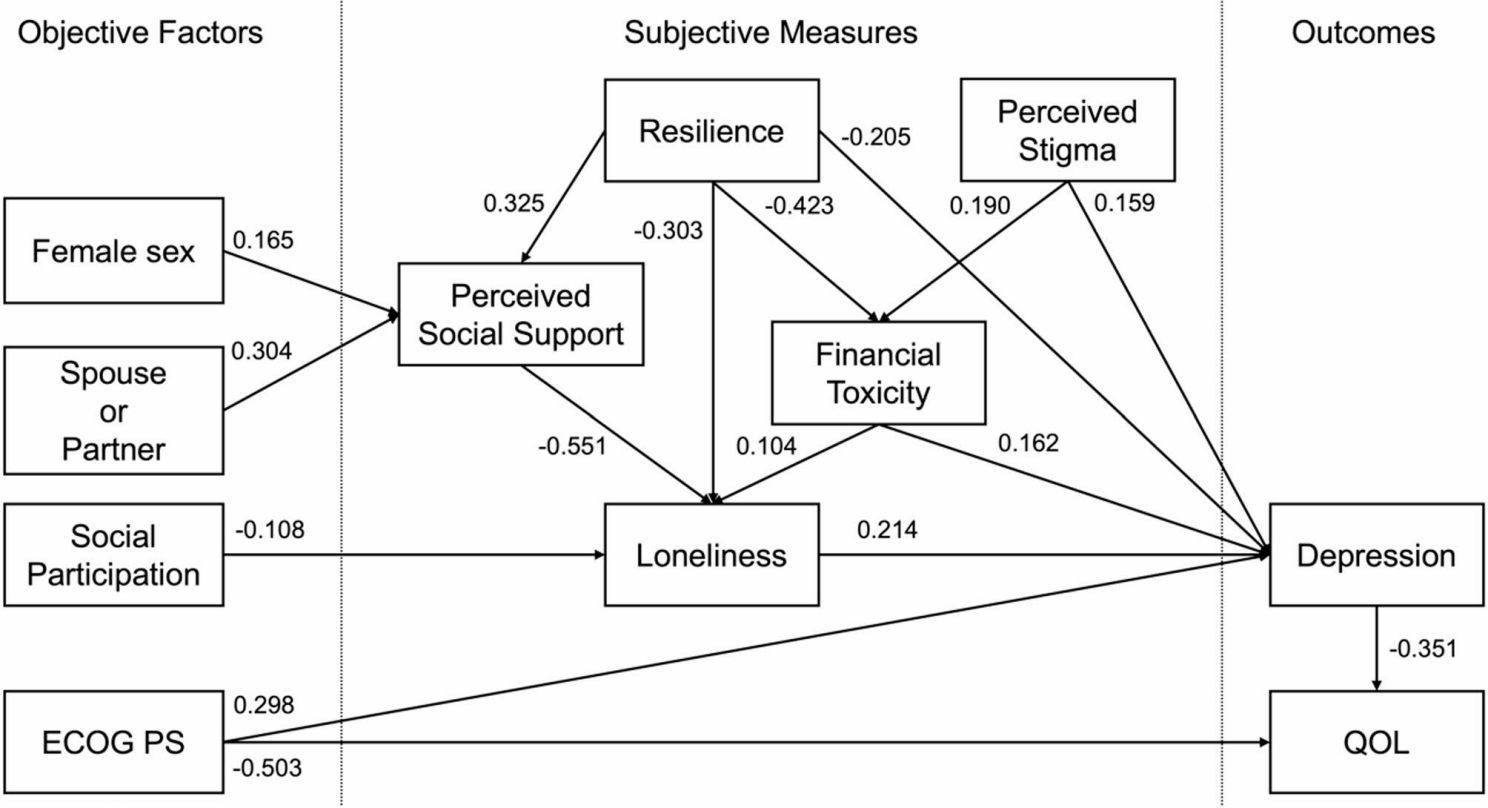
Structural equation model incorporating the association between loneliness and depression. PS, performance status; QOL, quality of life

Figure 1 shows the structural equation model based on the entire sample (*N* = 3,565), and Table 3 summarizes the standardized regression coefficients. The model showed a significant association between loneliness and depression (standardized coefficient = 0.214; 95% CI = 0.184 to 0.243; *P* < 0.001). PSS, resilience, financial toxicity, and stigma were directly or indirectly associated with loneliness and depression. Among the objective factors, female sex and the presence of a spouse or partner were associated with PSS, while non-job-related social participation was associated with loneliness. QOL was influenced by depression and performance status, and performance status was also directly associated with depression.

**Table 3:** Standardized regression coefficients for the structural equation model

Dependent	Independent	Standardized regression coefficient (95% confidence interval)	*P*-value
Perceived Social Support	Resilience	0.325 (0.298 to 0.353)	< 0.001
Perceived Social Support	Spouse or Partner	0.304 (0.277 to 0.332)	< 0.001
Perceived Social Support	Female Sex	0.165 (0.136 to 0.194)	< 0.001
Financial Toxicity	Perceived Stigma	0.190 (0.162 to 0.219)	< 0.001
Financial Toxicity	Resilience	−0.423 (−0.449 to −0.397)	< 0.001
Loneliness	Perceived Social Support	−0.551 (−0.571 to −0.531)	< 0.001
Loneliness	Financial Toxicity	0.104 (0.080 to 0.127)	< 0.001
Loneliness	Resilience	−0.303 (−0.327 to −0.278)	< 0.001
Loneliness	Social Participation	−0.108 (−0.129 to −0.087)	< 0.001
Depression	Loneliness	0.214 (0.184 to 0.243)	< 0.001
Depression	Financial Toxicity	0.162 (0.134 to 0.191)	< 0.001
Depression	Perceived Stigma	0.159 (0.133 to 0.185)	< 0.001
Depression	Resilience	−0.205 (−0.237 to −0.173)	< 0.001
Depression	ECOG performance status	0.298 (0.274 to 0.322)	< 0.001
Quality of Life	Depression	−0.351 (−0.375 to −0.327)	< 0.001
Quality of Life	ECOG performance status	−0.503 (−0.525 to −0.482)	< 0.001

Other variables were excluded because they reduced the model’s fitness. Among cancer types, only esophageal cancer showed a significant association with loneliness in multivariable linear regression (β = −1.17; 95% CI = −2.29 to −0.04; *P* = 0.042); however, the small effect size (1.17 points on the 0–80 scale) resulted in its exclusion from the final model.

The fit indices in the final model were as follows: CFI = 0.956, GFI = 0.961, AGFI = 0.892, and RMSEA = 0.074, indicating a moderate-to-good fit. Although additional pathways and latent variables were explored, none improved the fit, making this the final model.

### Comparison between AYA and non-AYA individuals

Multi-group analysis showed a stronger association between loneliness and depression in the AYA group (standardized coefficient = 0.315; 95% CI = 0.256 to 0.374; *P* < 0.001) than in the non-AYA group (standardized coefficient = 0.209; 95% CI = 0.175 to 0.243; *P* < 0.001), with no overlap in the CIs (Table 4). The associations between resilience and PSS, resilience and depression, and performance status and depression also showed no overlap in CIs between the two groups.

**Table 4:** Coefficient comparison through multi-group analysis

Dependent	Independent	Coefficient in AYAs	Coefficient in non-AYAs	Overlap
Perceived Social Support	Resilience	0.226 (0.162 to 0.289)	0.342 (0.311 to 0.372)	None
Perceived Social Support	Spouse or Partner	0.237 (0.173 to 0.300)	0.317 (0.286 to 0.347)	
Perceived Social Support	Female Sex	0.214 (0.149 to 0.278)	0.160 (0.128 to 0.193)	
Financial Toxicity	Perceived Stigma	0.160 (0.095 to 0.224)	0.165 (0.132 to 0.198)	
Financial Toxicity	Resilience	−0.376 (−0.434 to −0.317)	−0.424 (−0.454 to −0.395)	
Loneliness	Perceived Social Support	−0.514 (−0.562 to −0.466)	−0.571 (−0.592 to −0.549)	
Loneliness	Financial Toxicity	0.124 (0.068 to 0.180)	0.092 (0.066 to 0.118)	
Loneliness	Resilience	−0.280 (−0.336 to −0.224)	−0.301 (−0.328 to −0.274)	
Loneliness	Social Participation	−0.122 (−0.174 to −0.070)	−0.090 (−0.114 to −0.067)	
Depression	Loneliness	0.315 (0.256 to 0.374)	0.209 (0.175 to 0.243)	None
Depression	Financial Toxicity	0.155 (0.096 to 0.214)	0.172 (0.139 to 0.204)	
Depression	Perceived Stigma	0.161 (0.105 to 0.217)	0.089 (0.058 to 0.119)	
Depression	Resilience	−0.089 (−0.153 to −0.026)	−0.253 (−0.290 to −0.217)	None
Depression	ECOG performance status	0.376 (0.326 to 0.427)	0.246 (0.218 to 0.274)	None
Quality of Life	Depression	−0.319 (−0.375 to −0.264)	−0.316 (−0.343 to −0.289)	
Quality of Life	ECOG performance status	−0.496 (−0.546 to −0.445)	−0.529 (−0.553 to −0.506)	

*AYA* Adolescents and young adults

Furthermore, a chi-square difference test comparing a model with equal regression coefficients for the loneliness-to-depression pathway between groups to an unconstrained model showed a significant difference in fitness (χ²(1) = 29.51, *P* < 0.001), indicating that these coefficients differed significantly between the AYA and non-AYA groups.

## Discussion

This study used cross-sectional data from an online survey, which included various psychosocial scales, and developed a structural equation model to examine the association between loneliness and depression among cancer survivors. The model revealed a link between loneliness and depression, with other psychosocial factors directly or indirectly influencing this relationship. As hypothesized, group comparisons revealed that the association between loneliness and depression was stronger among AYAs than among non-AYAs.

The stronger association between loneliness and depression was observed among AYA cancer survivors than among non-AYA individuals, based on a model developed using a large sample and incorporating various psychosocial factors. While the relationship between loneliness and depression aligns with previous studies [11, 17–20], the generational difference in the strength of this association has not been examined in AYA cancer survivors and has not been observed among the general population [6, 31]. Thus, the finding adds to the literature by implying that the impact of cancer diagnosis or treatment on loneliness and depression can be particularly substantial in AYA populations. This may be related to findings generally observed in this age group, including social instability [7], heightened loneliness [32], highly prevalent mental illness [33], and suicide as the leading cause of death in Japan [34]. These suggest that loneliness may serve as a modifiable mediator for the prevention or treatment of depression, particularly among AYA cancer survivors. Addressing loneliness may also contribute to suicide prevention, given the critical role of depression management in reducing suicide risk [35].

A meta-analysis indicated that interventions focusing on social skills, emotional skills, social interactions, and social support alleviate loneliness among younger individuals [36]. In Japan, interventions tailored to AYAs, such as a behavioral activation smartphone app for young breast cancer survivors, have reportedly reduced fear of recurrence [37]. Similarly, a problem-solving smartphone app designed to reduce distress among AYAs is under development [38]. Expanding such interventions to address loneliness among AYA cancer survivors would be beneficial.

The relationships between loneliness and psychosocial factors, including PSS, resilience, and financial toxicity, were consistent with previous findings [11, 17–20]. Notably, resilience emerged as a key factor that directly influenced social support, financial toxicity, and depression. The model also showed an indirect effect of cancer stigma on loneliness, mediated by financial toxicity, which may have resulted from workplace disadvantages due to stigma toward individuals with cancer [39]. In addition to individual-level interventions, societal efforts to reduce cancer-related stigma would be essential to alleviate loneliness and depression.

Among objective factors, the presence of a spouse or partner indirectly influenced loneliness through PSS. In addition, social participation, such as volunteering, was directly associated with loneliness. These objective factors should be assessed as potential risk indicators in clinical settings. Given its potential for modification, social participation may serve as an intervention target. A meta-analysis demonstrated the efficacy of interventions focused on social interaction [36]. Emerging approaches, such as virtual reality-based peer support designed to alleviate loneliness [40], may also be particularly effective for AYA cancer survivors who tend to be more comfortable with digital platforms.

This study has several limitations. First, the cross-sectional design prevented the determination of causal relationships. Longitudinal studies are required to validate the structural equation model developed in this study. Second, recruitment was conducted through a web-based survey with self-reported cancer-related information, such as type and stage. Data from participants recruited through medical institutions may be required for validation. Third, the online survey may have recruited relatively healthy and active individuals, which may have affected the pathways. Finally, the study did not include individuals without cancer, preventing evaluation of the impact of cancer diagnosis or treatment on the observed pathways.

## Conclusions

The present study developed a structural equation model using psychosocial measurements from cancer survivors, revealing a significant association between loneliness and depression, with a stronger coefficient observed among AYA participants. These findings suggest that loneliness among AYA cancer survivors may serve as a modifiable mediator for interventions aimed at preventing depression. This study also highlights several psychosocial variables that can aid in screening high-risk individuals and identifying intervention targets, warranting future longitudinal and intervention research.

## Data Availability

The datasets and codes used in this study are available from the corresponding author upon reasonable request.

## Abbreviations

AYA: Adolescent and young adult
QOL: Quality of life
PSS: Perceived social support
CFI: Comparative fit index
GFI: Goodness of fit index
AGFI: Adjusted goodness of fit index
RMSEA: Root mean square error of approximation
CI: Confidence interval
